# Acoustic Vector Sensor-Based UAV Sound Source Localization via Covariance Enhancement and Confidence Guidance Tracking

**DOI:** 10.3390/s26154716

**Published:** 2026-07-24

**Authors:** Jiayu Hou, Tianlun He, Da Chen

**Affiliations:** 1 School of Safety Science and Engineering, Civil Aviation University of China, Tianjin 300300, China; 2023095024@cauc.edu.cn; 2 School of Precision Instrument and Opto-Electronics Engineering, Tianjin University, Tianjin 300072, China; lunqiqi@yeah.net; 3 Tianjin Engineering Research Center of Civil Aviation Energy Environment and Green Development, Civil Aviation University of China, Tianjin 300300, China

**Keywords:** acoustic vector sensor, UAV localization, direction-of-arrival estimation, MUSIC, adaptive diagonal loading, confidence-weighted tracking

## Abstract

Unauthorized unmanned aerial vehicle (UAV) intrusions in sensitive areas such as airports have made accurate UAV detection and localization a pressing need. Acoustic sensing is passive and weather-independent, but conventional microphone arrays require many elements and a large aperture. This paper proposes an acoustic vector sensor (AVS)-based method, termed Covariance Enhancement and Confidence-guided Tracking for 3D Acoustic Localization (CECT-3DAL). A single AVS measures the sound pressure and three-axis particle velocity at one point. Adaptive diagonal loading improves the robustness of the covariance matrix at a low signal-to-noise ratio (SNR). An exponential spectral enhancement strategy sharpens the spatial spectrum peaks for direction estimation, and an eigenvalue-ratio-based confidence drives confidence-weighted smoothing of the angle sequences. Meanwhile, a dual-sensor geometric model provides a closed-form three-dimensional solution. In simulations, the azimuth and elevation root-mean-square errors (RMSEs) were below 1.5° for SNR above 4 dB. In an anechoic chamber, confidence-weighted smoothing reduced the azimuth and elevation standard deviations from 4.34° and 2.63° to 1.46° and 0.86°. In field experiments, the hovering azimuth stayed within a 90% span of 2–3.5°, with an average horizontal RMSE of 0.209 m against a GPS reference, and trajectories under various flight modes remained continuous and smooth. The proposed method offers a compact, passive, and low-cost solution for counter-UAV acoustic surveillance.

## 1. Introduction

In recent years, the rapid development of Unmanned Aerial Vehicle (UAV) technology and the continuous expansion of its application have enabled UAVs to play an increasingly important role in fields such as airport operations, urban management, logistics and agriculture [1,2,3]. However, the widespread deployment of UAVs has also introduced considerable safety concerns [4]. In particular, in sensitive areas such as airports, unauthorized UAV intrusions have become a severe challenge for aviation safety management worldwide [5]. Such intrusions can disrupt normal airport operations, endanger aircraft during take-off and landing, and may even lead to serious aviation accidents [6]. The growing frequency of these incidents has made counter-UAV (C-UAS) technology a research focus for both civil aviation regulators and the academic community [7]. Consequently, the effective and accurate detection and localization of unauthorized UAVs has become a key technical challenge in airport security and management.

Current technologies for detecting UAV intrusions fall mainly into three categories: radar, optical imaging, and radio frequency (RF) spectrum analysis [8,9]. In airport settings, however, each of these faces significant limitations. Radar, though generally effective for UAV detection, is hampered near airports by the strong reflections generated by aircraft during take-off and landing, which readily induce false alarms and missed detections. The complex buildings and ground infrastructure further introduce multipath reflections that complicate target identification [10]. Although recent radar studies have improved small UAV detection through multistatic micro-Doppler measurements [11] and CNN-based discrimination of drones from birds [12], the multipath problem in airport environments remains unresolved. Optical imaging performs well under favorable conditions but is highly sensitive to illumination and weather, exhibiting markedly degraded performance at night or in haze and rain [13]. Although deep learning and thermal infrared detectors have improved optical performance, poor visibility remains a fundamental limitation [14]. RF spectrum analysis depends on the communication link between the UAV and its operator; for UAVs operating autonomously, this link is weak or absent, undermining RF-based methods, while the dense radio activity surrounding airports further interferes with UAV signals and degrades localization accuracy [15]. Deep learning-based RF identification has been proposed to enhance robustness [16], yet it cannot detect UAVs that emit on RF signals at all.

Compared with the aforementioned methods, acoustic sensing provides an efficient means of monitoring and tracking UAVs. As a passive modality, it does not interfere with the normal operation of other equipment, and it remains effective even for UAVs flying autonomously without an active RF link. It has therefore been studied extensively. Existing work mainly recognizes the distinctive audio signatures of UAVs and estimates their direction of arrival (DOA) using compact sensor arrays. Yang et al. employed a deep learning model to construct a UAV surveillance system with multiple acoustic nodes [17]. Salman et al. proposed an efficient UAV monitoring method based on acoustic signatures [18]. Itare et al. used a genetic algorithm to optimize the microphone array geometry and localized UAVs acoustically by means of beamforming [19]. Blanchard et al. extracted UAV acoustic features through zero-phase selective band-pass filtering of the emitted sound and achieved acoustic localization and tracking of a multi-rotor UAV with only a few microphones [20]. Further studies have estimated DOA using microphone arrays mounted on UAVs [21,22], compared localization techniques across frequency bands [23], explored MEMS microphone-based passive localization [24], and investigated distributed microphone arrays [25]. However, existing acoustic detection systems generally require a large number of array elements, which entails complex installation and a large physical footprint.

The acoustic vector sensor (AVS) offers a promising way to overcome these limitations. An AVS senses the acoustic pressure and particle velocity simultaneously at a single point under free-field conditions [26]. Even under complex background noise, it can acquire the dipole directional particle velocity of the target source, and its directivity is frequency-independent over a wide frequency band [27], enabling accurate three-dimensional (3D) localization with greater degrees of freedom and flexibility in DOA estimation [28]. Building on these properties, various AVS-based DOA methods have been developed, including Capon-type estimators [29], multimodal multi-source detection [30], sparse recovery-based two-dimensional estimation [31], coprime vector sensor arrays [32], and methods robust to anisotropic noise [33]. However, most of these methods address underwater or stationary sources rather than in-air, low-altitude, slow-moving UAVs under strong outdoor noise. To fill this gap, this paper proposes a high-accuracy 3D UAV sound-source localization method based on AVS, termed CECT-3DAL, which constructs a 3D vector measurement model to enhance sensitivity to the directional information of the target source.

This paper further develops a subspace direction estimation algorithm based on covariance structure enhancement and confidence guided optimization. To address the instability of covariance matrix estimation caused by noise interference, an adaptive diagonal loading mechanism is first introduced, which dynamically adjusts the loading factor to enhance the eigenstructure of the covariance matrix and thereby improves the robustness of direction estimation under low signal-to-noise ratio (SNR) conditions. Then, by exploiting the main-lobe-to-side-lobe energy contrast of the spatial spectrum, an exponential spectral enhancement strategy is applied to sharpen the spectral peaks and facilitate azimuth and elevation peak extraction. To improve dynamic trajectory tracking, a localization confidence metric based on the eigenvalue ratio is constructed in the temporal domain, and confidence-weighted smoothing is applied to the estimated azimuth and elevation sequences to suppress transient outliers. Finally, the smoothed angles are fed into a dual-sensor spatial geometry model to obtain a closed-form solution for the UAV’s 3D position. Simulations, anechoic chamber tests, and outdoor field experiments demonstrate that, even with a limited number of sensors and complex background interference, the proposed method achieves accurate and stable 3D direction estimation and trajectory tracking of low-altitude, slow-moving UAVs.

## 2. Materials and Methods

The acoustic source of a UAV is typically located in the far field relative to the receiving sensor, and the background noise in practical environments is complex. In isotropic ambient noise, the acoustic pressure and particle velocity are uncorrelated or only weakly correlated, whereas for the target signal they are strongly correlated; this distinction provides a basis for extracting the target under complex noise [34]. By acquiring both pressure and particle velocity, an AVS provides richer sound field information than a scalar sensor: at a single spatial point, it measures not only the omnidirectional scalar pressure but also the particle velocity along three orthogonal axes, which carries directional information [35]. Consequently, the multi-channel output of an AVS affords greater flexibility for vector-array beamforming and direction estimation, together with improved spatial resolution and strong interference rejection compared with scalar sensor arrays of comparable size, enabling accurate identification and localization of acoustic sources in complex acoustic environments.

When the source emits a uniform plane-wave field and the signal is band-limited, the sound field obeys the linearized Euler equation. For a plane-wave field, the relationship between the particle velocity and the acoustic pressure can be expressed as

(1) $$\frac{\partial v}{\partial t}=-\frac{1}{\rho_{0}}\nabla p$$

where ***v*** is the particle velocity, p is the acoustic pressure, and $\rho_{0}$ is the medium density. For a harmonic acoustic wave, the pressure and particle velocity satisfy

(2) $$p(t)=P_{0}e^{j\left(\omega t+\phi_{1}\right)}$$

(3) $$v(t)=V_{0}e^{j\left(\omega t+\phi_{2}\right)}$$

where $P_{0}$ and $V_{0}$ are the pressure and velocity amplitudes, and $\phi_{1}$ and $\phi_{2}$ are the corresponding initial phases. A 3D AVS can simultaneously measure, at a single point, the particle velocity components along the *x*, *y*, and *z* axes:

(4) $$v=\left[v_{x},v_{y},v_{z}\right]$$

The direction vector of the source signal received by the sensor can be expressed as

(5) $$u=\left(\cos\theta \cos\varphi ,\sin\theta \cos\varphi ,sin\varphi \right)$$

where θ is the azimuth angle, and φ is the elevation angle. From (1) and (5), the relationship between the pressure signal and the particle velocity can be written as

(6) $$v(t)=-\frac{p\left(t\right)}{\rho_{0}c}u$$

Let $y_{p}\left(t\right)$ be the pressure signal received by the sensor at time *t* and $n_{p}\left(t\right)$ be the noise component; then, the pressure measurement of the vector sensor can be expressed as

(7) $$y_{p}\left(t\right)=p\left(t\right)+n_{p}\left(t\right)$$

Similarly, the particle velocity measurement can be expressed as

(8) $$y_{v}\left(t\right)=v\left(t\right)+n_{v}\left(t\right)$$

Thus, the signal received by a single AVS can be expressed as

(9) $$x\left(t\right)=\left[\begin{array}{c} y_{p}(t)\\ y_{v}(t) \end{array}\right]=\left[\begin{array}{c} 1\\ u \end{array}\right]p\left(t\right)+\left[\begin{array}{c} n_{p}(t)\\ n_{v}(t) \end{array}\right]$$

Accordingly, for an incident signal *s*(*t*), the output of a single vector sensor element is

(10) $$x_{v}\left(t\right)=a_{v}\left(\theta_{0},\varphi_{0}\right)s\left(t\right)+n_{v}(t)$$

where $a_{v}\left(\theta_{0},\varphi_{0}\right)$ is the steering vector.

For a single AVS consisting of one acoustic-pressure channel and three orthogonal particle velocity channels, the combined four-channel output in the presence of L incident sources can be expressed as

(11) $$X\left(t\right)=\sum_{i=1}^{L}a\left(\theta_{i},\varphi_{i}\right)s_{i}\left(t\right)+n\left(t\right)\;$$

By averaging the outer products of the snapshots over a sufficiently long time window, the sample covariance matrix can be estimated as

(12) $$R=\frac{1}{T}\sum_{t=1}^{T}X(t){X(t)}^{H}\;$$

To enhance the stability of the covariance matrix under low-SNR conditions, an adaptive diagonal loading technique is introduced:

(13) $$R_{load}=R+\alpha \frac{tr(R)}{M}I$$

where tr(R) is the trace of R, *M* is the dimension of the covariance matrix for the four-channel AVS output. The loading factor α is adjusted dynamically according to the local SNR

(14) $$\alpha =\alpha_{base}\cdot min(1,\frac{P_{n}}{P_{t}})\;$$

where $P_{n}$ denotes the estimated noise-floor RMS, $P_{t}$ denotes the RMS of the current frame, and $\alpha_{base}$ is the base loading level determined empirically. The loaded covariance matrix is then eigen-decomposed as:

(15) $$R_{load}=E\Lambda E^{H}\;$$

where $E=\left[e_{1},\;e_{2},e_{3},e_{4}\right]$ is the eigenvector matrix and $\Lambda =diag[\lambda_{1},\lambda_{2},\lambda_{3},\lambda_{4}]$ is the diagonal matrix of eigenvalues. The noise subspace is

(16) $$E_{n}=[e_{2},e_{3},e_{4}]\;$$

Under the single dominant source assumption adopted in this study, the eigenvector corresponding to the largest eigenvalue spans the signal subspace, while the remaining three eigenvectors form the noise subspace.

The DOA of the incident signal can be estimated by enhancing the peak of the spatial spectrum-enhanced MUSIC (SPM-MUSIC)spatial spectrum, that is,

(17) $$P_{SPM-MUSIC}\left(\theta ,\varphi \right)=\frac{1}{{\left(\left|a{\left(\theta ,\varphi \right)}^{H}E_{n}{E_{n}}^{H}a\left(\theta ,\varphi \right)\right|\right)}^{q}+\varepsilon }\;$$

where *q* > 1 is the exponential enhancement factor, and ε is a small positive constant introduced to avoid division by zero. A two-dimensional grid search is performed over the predefined azimuth–elevation domain. When the candidate steering vector matches the true incident direction, it becomes approximately orthogonal to the noise subspace, producing a sharp spectral peak. The direction-of-arrival estimate is obtained from the maximum of the SPM-MUSIC spectrum, yielding $\left(\hat{\theta },\hat{\varphi }\right)$.

**Figure 1:**
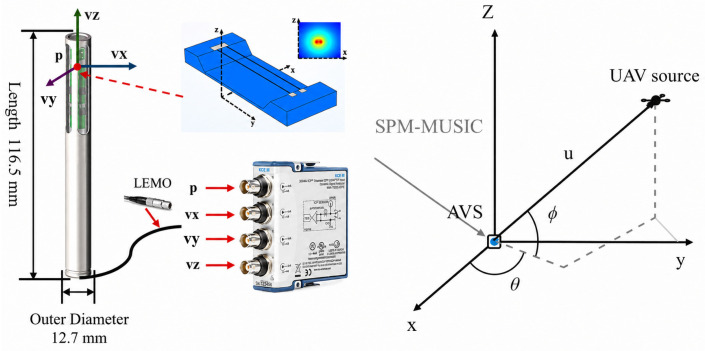
Integrated three-dimensional acoustic vector sensor and single-AVS DOA estimation principle.

The sensing principle and hardware configuration of the single-AVS-based UAV acoustic direction estimation system are illustrated in Figure 1.

As shown in Figure 1, an integrated three-dimensional acoustic vector sensor (AVS) was used in this study. The sensor integrates one acoustic pressure sensing unit and three orthogonally arranged particle velocity sensing units, so that the sound pressure p and the three particle-velocity components vx, vy, and vz can be synchronously acquired at the same measurement point [36]. The sensor has a slender cylindrical integrated structure, with an outer diameter of 12.7 mm, a length of 116.5 mm, and a mass of approximately 37.8 g. It is connected to a dedicated signal conditioner through a 7-pin LEMO connector. The pressure channel is a conventional acoustic pressure microphone, while the velocity channels are hot-wire particle velocity sensors, jointly forming a composite AVS. The pressure channel has a frequency response of 20–10,000 Hz, and the velocity channels have a frequency response of 10–10,000 Hz. At 1 kHz, the nominal sensitivities of the pressure and velocity channels are both 50 mV, with signal-to-noise ratios of approximately 65 dB and 55 dB, respectively.

In practice, the azimuth and elevation angles estimated by a single AVS are inevitably affected by background noise, reflections, occlusion, and other factors, causing the frame-by-frame DOA estimates to fluctuate over time. Therefore, an eigenvalue ratio-based confidence metric and a confidence-weighted smoothing mechanism are introduced to improve the stability of the estimated angular trajectory.

In each frame, a covariance matrix is constructed from the four-channel output of the single AVS, and its two largest eigenvalues, $\lambda_{1}(t)$ and $\lambda_{2}(t)$, are obtained. The per-frame eigenvalue ratio (EVR) is used as the confidence measure:

(18) $$EVR\left(t\right)=\frac{\lambda_{1}(t)}{\lambda_{2}\left(t\right)+\varepsilon }$$

The normalized EVR is used as the confidence of the current DOA estimate:

(19) $$conf\left(t\right)=min(1,\frac{EVR(t)}{\gamma })$$

where γ is the normalization threshold. The azimuth and elevation are then smoothed with confidence weighting:

(20) $$\hat{\theta_{\left(t\right)}}=\frac{\sum_{k=t-K}^{t+K}w(k)\theta_{i}(k)}{\sum_{k=t-K}^{t+K}w(k)}$$

(21) $$\hat{\varphi_{\left(t\right)}}=\frac{\sum_{k=t-K}^{t+K}w(k)\varphi_{i}(k)}{\sum_{k=t-K}^{t+K}w(k)}$$

where $w\left(k\right)=conf\left(k\right)+\varepsilon$, K denotes the half-width of the smoothing window, and ε prevents a zero weighting factor.

## 3. Experiments and Results

This section validates the proposed algorithm through computer simulations, anechoic chamber tests, and outdoor field experiments. The simulations and chamber tests correspond to free field conditions with reflections removed, whereas the field experiments examine the method under realistic outdoor noise. The simulations evaluate the DOA estimation accuracy under different SNRs and integration times, the 3D localization of fixed sources, and the resolution of multiple sources so as to comprehensively assess the performance of the AVS and the algorithm and to provide a basis for the subsequent real-data experiments.

### 3.1. Computer Simulation

The localization accuracy improves to some extent as the number of AVSs in the array increases; however, given the miniaturization and real-time requirements of the UAV localization system, localization should be accomplished with as few sensors as possible. Accordingly, a localization simulation was carried out with a single AVS, and the 3D localization performance was simulated in Python 3.13.

To evaluate the DOA estimation accuracy at different SNRs, the source was placed at 45° and 60° in the simulation, and the SNR was defined as

(22) $$SNR=10\mathrm{lg}\left(\frac{S}{N}\right)$$

where S and N denote the signal power and the noise power, respectively. The error metrics for each experiment group were defined as

(23) $$RMSE\left(\theta \right)=\sqrt{\frac{1}{J}\sum_{j=1}^{J}{\left({\hat{\theta }}_{j}-\theta_{0}\right)}^{2}}$$

(24) $$RMSE\left(\varphi \right)=\sqrt{\frac{1}{J}\sum_{j=1}^{J}{\left({\hat{\varphi }}_{j}-\varphi_{0}\right)}^{2}}$$

**Figure 2:**
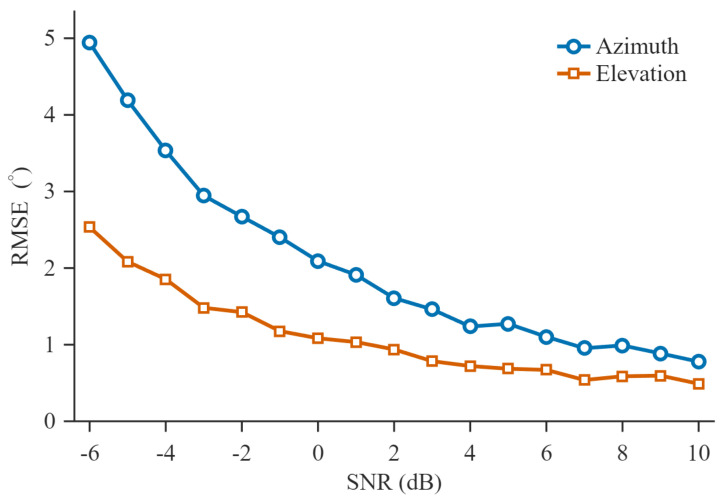
RMSE versus SNR.

The DOA estimation accuracy under different SNRs is shown in Figure 2.

Figure 2 shows that the DOA estimation accuracy for the UAV target improves as the SNR increases; under the given integration time, when the SNR exceeds 4 dB, the root-mean-square errors of both the azimuth and the elevation are below 1.5°.

**Figure 3:**
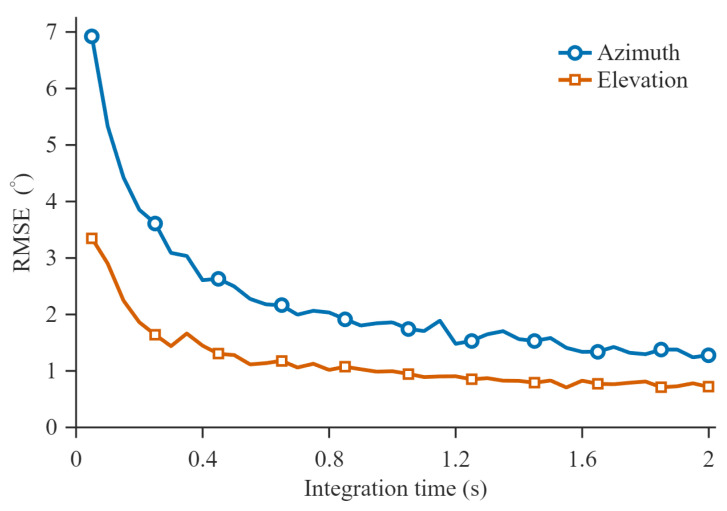
RMSE versus integration time.

With the SNR fixed at 10 dB, the DOA estimation accuracy under different integration times is shown in Figure 3.

Figure 3 shows that the azimuth estimation error decreases rapidly with increasing integration time and then levels off. When the integration time exceeds 0.9 s, the azimuth error falls below 2° and the elevation error falls below approximately 1°, with only marginal improvement thereafter. This reflects a trade-off between estimation accuracy and real-time performance.

**Figure 4:**
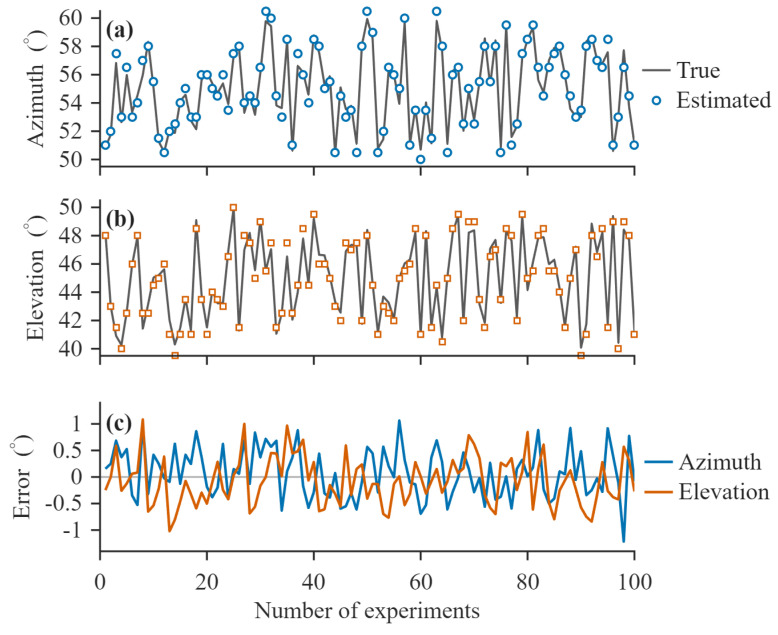
Localization results and error analysis for a fixed source. (**a**) comparison between the true and estimated azimuth angles; (**b**) comparison between the true and estimated elevation angles; (**c**) corresponding azimuth and elevation estimation errors.

A localization simulation was carried out for a fixed source in a 3D free field. First, 100 source points were randomly generated within an azimuth range of 50–60° and an elevation range of 40–50°, with white Gaussian noise added at an SNR of 10 dB. This Monte Carlo evaluation assessed the robustness of the algorithm under randomized source positions. Figure 4a and Figure 4b show the comparison between the true and estimated azimuth and elevation angles for the 100 randomly generated source positions, respectively, and Figure 4c presents the corresponding estimation errors.

As shown in Figure 4, the proposed SPM-MUSIC method achieved accurate DOA estimation using a single AVS, with RMSEs of 0.47° in azimuth and 0.46° in elevation, and maximum absolute errors within 1.3°. Compared with the elevation angle, the azimuth angle exhibited a slightly larger estimation error. This can be attributed to the reduced horizontal particle velocity components at relatively high elevation angles, since the horizontal projection is proportional to cosφ, which weakens the observability of the azimuth.

**Figure 5:**
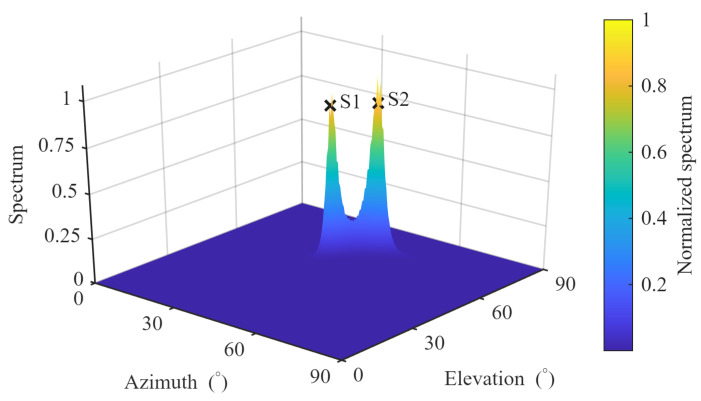
SPM-MUSIC spatial spectrum for two sources.

To verify the multi-source resolving capability of the proposed algorithm, two sources, S1 (36°, 60°) and S2 (45°, 70°), were placed in the free field. The resulting normalized SPM-MUSIC spatial spectrum is shown in Figure 5. The spectrum forms two distinct, sharp, and well-separated peaks at the true directions of the two sources, and the peak locations agree with the preset directions. This demonstrates that the proposed method can effectively resolve two sources with closely spaced azimuth angles, indicating good multi-source resolution capability.

Overall, the simulations confirm that a single compact AVS, combined with the proposed CECT-3DAL processing, provides accurate and robust DOA estimation across varying SNR and integration times, for both single and closely positioned sources. These results establish a baseline under ideal conditions, which the following subsections test on real acoustic signals in an anechoic chamber and in outdoor field experiments.

### 3.2. Anechoic Chamber Experiment

**Figure 6:**
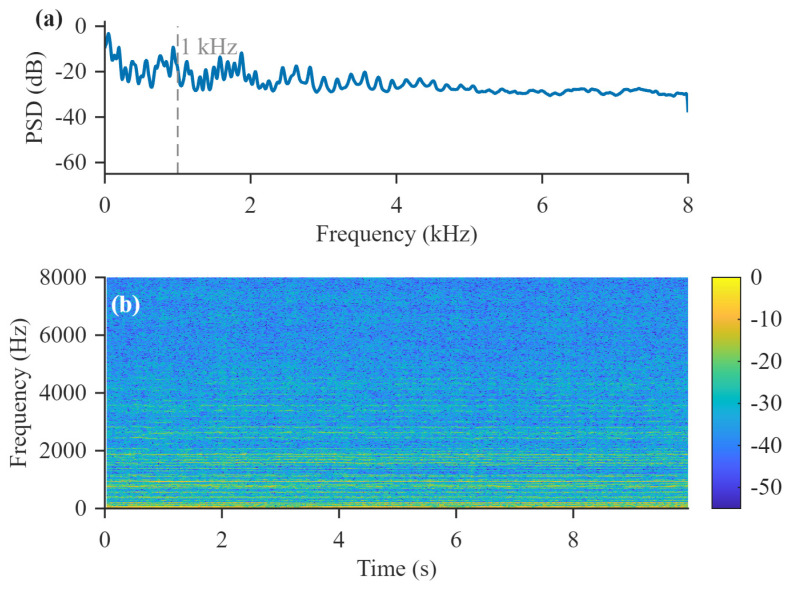
Measured noise characteristics of the UAV in flight: (**a**) power spectral density, with the dashed line marking 1 kHz; (**b**) spectrogram of the 0–8000 Hz band.

After its feasibility was verified by computer simulation, the algorithm was further evaluated in an anechoic chamber, which provides reflection-free, near-free-field conditions for assessing its direction estimation performance on a real source. To make the test representative of the intended application, the source should reproduce the dominant spectral characteristics of UAV noise. For this purpose, the noise of a UAV in flight was first measured and analyzed to identify its dominant frequency band, which then guided the choice of the test source frequency. Figure 6 shows the measured noise spectrum of the UAV.

**Figure 7:**
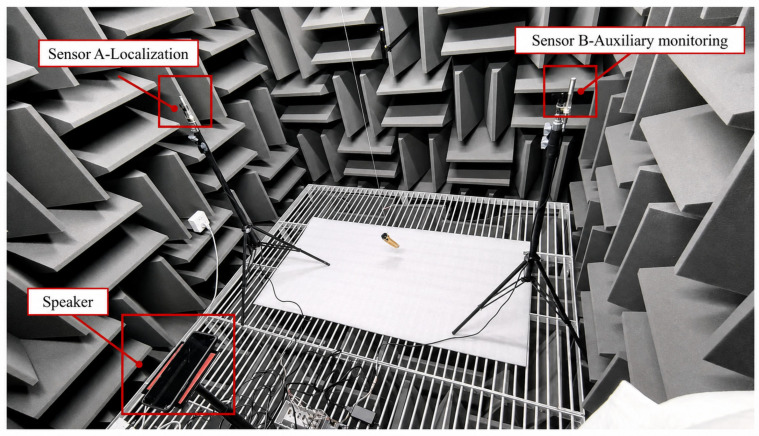
Experimental environment in the anechoic chamber.

Spectral analysis shows that the UAV noise energy is concentrated at low frequencies: the power spectral density in Figure 6a decreases markedly above about 2 kHz, with the strongest components below 1 kHz, while the spectrogram in Figure 6b exhibits dense harmonic lines below about 2 kHz, arising mainly from the rotation of the rotor blades. Accordingly, a 1 kHz tone, lying within this dominant band, was adopted as the test source. The four-channel signal was recorded with a single AVS at a sampling rate of 48 kHz. The experimental setup is shown in Figure 7.

The four AVS channels were conditioned by a KCE5 M four-channel signal conditioner and synchronously digitized using an NI cDAQ-9189 chassis equipped with a single NI-9234 four-channel acquisition module (National Instruments (ni.com), Austin, TX, USA). The module features a 24-bit analog-to-digital converter and a dynamic range of 102 dB, and the sampling rate was set to 48 kHz for the anechoic chamber experiment.

For the acquired raw vector acoustic signal, the algorithm first applied a band-pass filter over the 900–1200 Hz range and then performed short-time framing with a frame length of 0.1 s and a 50% inter-frame overlap, corresponding to about 4800 samples per frame with a hop of 2400 samples. To discard frames with weak energy or dominated by background noise, a dynamic background noise threshold criterion was applied: only frames whose RMS value exceeded 1.1 times the dynamic noise threshold proceeded to subsequent processing. Since the chamber was nearly free of ambient interference, the residual background noise originated almost entirely from the acquisition equipment and human operation.

The dynamic background noise threshold *N_k_* is given by

(25) $$N_{k}=\left(1-\lambda \right)N_{k-1}+\lambda E_{k}$$

where *E_k_* is the RMS value of the pressure signal in the *k*-th frame, and λ is the update factor, set to 0.02 in this experiment.

The recordings were then processed with the pipeline described in Section 2. The covariance matrix was estimated by applying a sliding average over 256-point subframes with a hop size of 128, with adaptive diagonal loading applied according to the per-frame SNR. The SPM-MUSIC spectrum was computed with q = 2.0, and the azimuth and elevation were taken from its dominant peak. A frame was retained as valid only when its eigenvalue ratio (EVR) exceeded 1.2. To facilitate comparison with the simulation performance curves, the source-to-sensor distances and estimated SNRs for the two anechoic-chamber measurements are summarized in Table 1.

The SNR was estimated from the pressure channel after band-limited spectral analysis. The target band energy was calculated over 900–1200 Hz, and the noise floor was estimated from the adjacent frequency bands of 600–850 Hz and 1250–1500 Hz.

**Figure 8:**
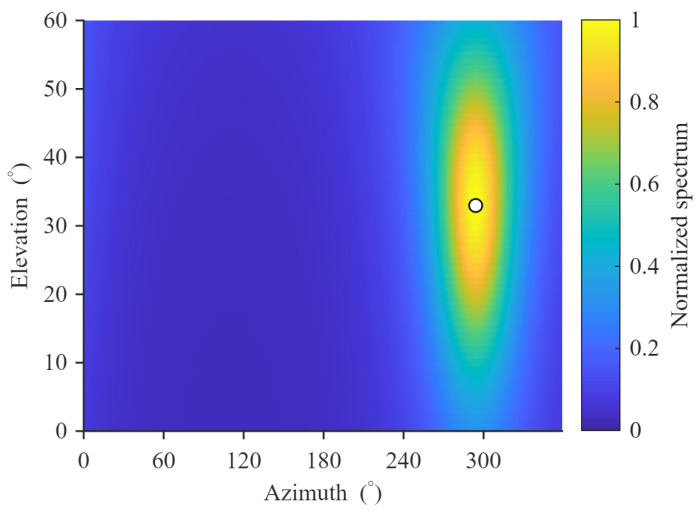
Normalized SPM-MUSIC spatial spectrum of a representative frame for the fixed source in the anechoic chamber. The white circle marks the spectral peak at approximately (293°, 33°).

Figure 8 shows the normalized SPM-MUSIC spatial spectrum of a representative frame for the fixed source. The spectrum exhibits a single sharp peak at approximately (293°, 33°), in agreement with the true source direction, and the side-lobe level remains low over the rest of the scanning plane, indicating that the exponential spectral enhancement effectively concentrated the spectral energy around the true direction.

**Figure 9:**
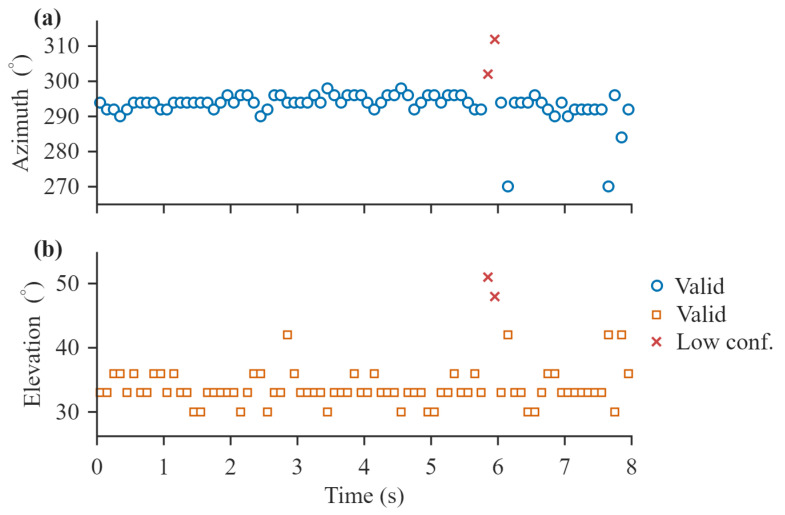
Raw per-frame DOA estimates for the fixed source in the anechoic chamber: (**a**) azimuth; (**b**) elevation. Red crosses denote low-confidence frames.

Figure 9 shows the raw per-frame DOA estimates obtained by SPM-MUSIC for the fixed source over a stable 8 s recording segment (80 frames in total). The red crosses denote low-confidence frames, and the average confidence over this segment is 0.604. Most frames yield stable azimuth and elevation estimates, while only a few outlier frames affected by transient interference are identified as low-confidence.

**Figure 10:**
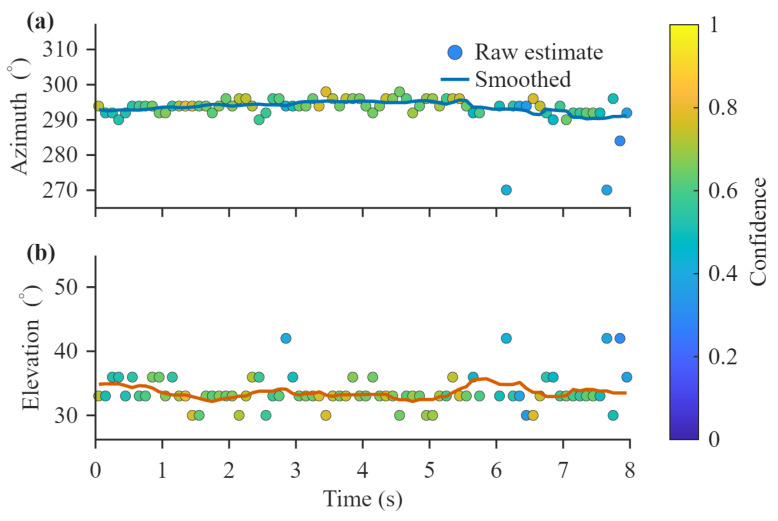
Results of confidence-weighted smoothing: (**a**) azimuth; (**b**) elevation. The scatter point color indicates the per-frame confidence, and the solid curves are the smoothed trajectories.

Figure 10 shows the results of confidence-weighted smoothing, where the scatter point color indicates the per-frame confidence and the curve is the smoothed direction trajectory. After smoothing, the standard deviation of the azimuth decreases from 4.34° to 1.46° and that of the elevation from 2.63° to 0.86°. The outlier frames in the raw estimates are effectively suppressed, yielding a smoother and more continuous trajectory. These results show that confidence-weighted smoothing effectively suppresses anomalous frames and markedly improves the temporal continuity and stability of the direction trajectory.

In summary, the anechoic chamber experiment results confirm that the proposed CECT-3DAL method yields stable and accurate direction estimates for a real source under free field conditions, and that confidence-weighted smoothing substantially improves the temporal continuity of the trajectory. This provides a basis for the subsequent field experiments under realistic outdoor conditions.

### 3.3. Field Experiment

The anechoic chamber experiment verified the method under controlled free field conditions. To assess its practicality in realistic environments—where wind, ambient noise, ground reflections, and UAV motion all coexist—an outdoor field experiment was further conducted using a real multi-rotor UAV as the target source. The field experiment was designed to evaluate the proposed method under short-range, low-altitude UAV acoustic tracking conditions. The elevation search range of the algorithm was set to −90° to +90°. In practical applications, however, the minimum reliable elevation angle and the maximum detectable range are not fixed sensor parameters but depend on the UAV acoustic radiation level, source-to-sensor distance, background noise, ground reflection, SNR, and the EVR-based confidence threshold.

**Figure 11:**
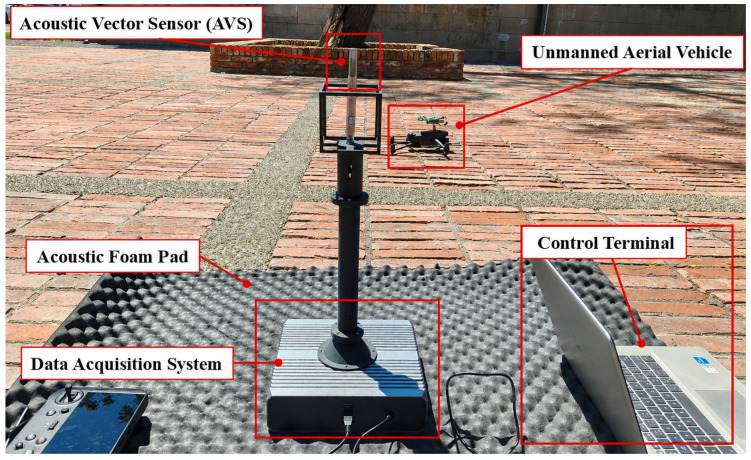
Setup of the outdoor UAV experiment.

Figure 11 shows the on-site setup of the outdoor UAV localization experiment. The system mainly comprised an AVS mounted on a stand, a data acquisition system, a control terminal (laptop), and a UAV serving as the target source. An acoustic foam pad was placed beneath the sensor base to suppress ground reflections and approximate free field conditions. The experiment was conducted in an open outdoor area, where the UAV performed hovering and horizontal-movement flights in the airspace above the sensor. The AVS captured the UAV’s radiated noise, which was recorded by the data acquisition system and processed by the control terminal for direction estimation and trajectory tracking of the UAV. It should be noted that aircraft takeoff and landing noise was not included in the field experiments because calibrated aircraft-engine recordings near active airport operations were not available under the required safety and permission constraints. Therefore, the present field tests focus on validating the proposed SPM-MUSIC-based direction estimation and confidence-guided tracking under real outdoor UAV acoustic conditions.

Owing to the saving mechanism of the acquisition software developed in Python 3.13, the audio was stored in segments of about 10 s. For long-duration experiments such as circling, adjacent segments were therefore stitched together according to the experiment log and trajectory continuity. The recordings were then processed with the same pipeline as in the anechoic chamber experiment. After mean removal and suppression of the 50 Hz power-line interference and its harmonics, the per-frame azimuth and elevation were estimated by the SPM-MUSIC algorithm with adaptive diagonal loading, screened by the EVR criterion, and refined by the proposed confidence-weighted smoothing. The GPS data, after outlier removal and smoothing, served as the reference for validating the estimated trajectories.

**Figure 12:**
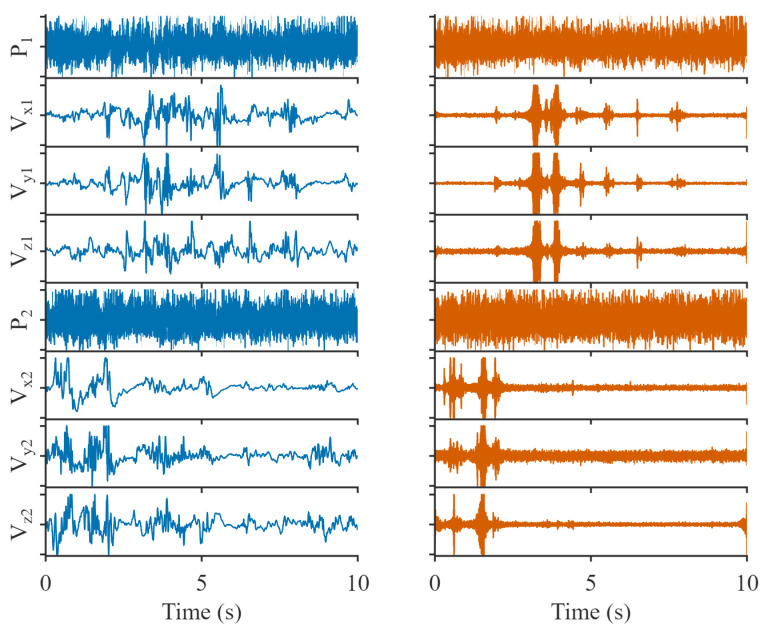
Typical four-channel signals independently recorded by two identical AVSs: (**left**) raw signals; (**right**) after 50 Hz power-line suppression and band-pass filtering. The top four channels correspond to sensor 1 and the bottom four to sensor 2.

Figure 12 shows typical four-channel signals recorded independently by two identical AVSs. In the raw signals (left column), the field recordings simultaneously contain the UAV sound, environmental noise, and low-frequency disturbances. The pressure channels exhibited a strong broadband background, while the amplitudes of the three velocity channels fluctuated with the changing relative direction of the UAV, reflecting the sensor’s sensitivity to directional information. After 50 Hz power-line suppression and band-pass filtering (right column), the low-frequency drift and the power-line component, together with its harmonics, were markedly attenuated. The envelope of the UAV sound became more prominent and the directional variations of the velocity components became clearer, thereby providing a more stable and reliable input for the subsequent SPM-MUSIC-based direction estimation.

**Figure 13:**
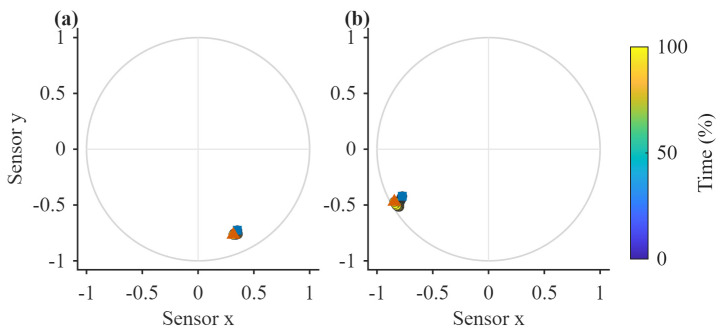
Directional stability in two hover experiments: (**a**) hover 1; (**b**) hover 2. The unit direction vector is projected onto the sensor x–y plane. Color indicates normalized time, and the square and triangle mark the start and end points, respectively.

Figure 13 shows the distribution of the estimated unit direction vector projected onto the sensor x–y plane for two hover experiments, where the color (from blue to yellow) indicates the normalized time progression. Both datasets correspond to a stable hover window of about 7.9 s. Throughout this interval, the direction vector remained tightly concentrated, with 90% azimuth spans of only 2.36° and 3.49°, respectively, indicating that the AVS yields highly stable and consistent estimates.

**Figure 14:**
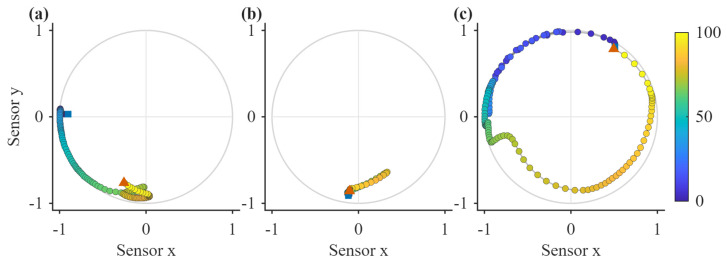
Unit direction-vector trajectories under three flight modes: (**a**) straight; (**b**) oblique; (**c**) circling. Color indicates normalized time, and the square and triangle mark the start and end points, respectively.

Figure 14 shows the estimated unit direction vector trajectories under three flight modes—straight, oblique, and circling—where the color (from blue to yellow) indicates time progression. The straight-flight segment (Figure 14a) lasted about 17.1 s, during which the direction vector traced a continuous arc in the x–y plane with a 90% azimuth span of about 96.6°, clearly capturing the linear fly-by of the UAV. The oblique segment (Figure 14b) lasted about 6.6 s, with the azimuth and elevation varying simultaneously and continuously, corresponding to the oblique climbing motion of the UAV. The circling experiment (Figure 14c), lasting about 56.5 s, formed a nearly closed ring in the sensor coordinate frame. These results demonstrate that the proposed CECT-3DAL method can continuously and stably track the directional changes of the UAV under different flight modes, yielding smooth trajectories consistent with the actual motion.

**Figure 15:**
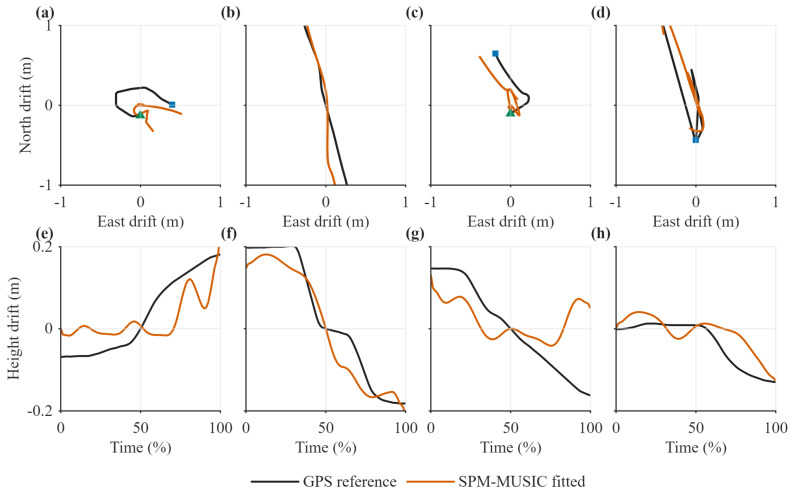
Consistency validation between the acoustic estimates and the GPS reference for four hover trials (B1–B4): (**a**–**d**) horizontal drift in the east–north plane; (**e**–**h**) height drift versus normalized time. The square and triangle mark the start and end points, respectively.

To further and quantitatively assess the hovering performance, the acoustic estimates were registered to the GPS reference for four hover trials (B1–B4). Since the audio and GPS data were not hardware-synchronized, a normalized time-shift correction was applied before comparison. Figure 15 shows the horizontal drift (a–d) and the height drift versus normalized time (e–h) for the four trials. In all cases, the trajectory reconstructed from the proposed method and registered to the GPS reference follows the reference closely, with the horizontal drift confined within about ±0.5 m and the height drift within ±0.2 m. The mean horizontal RMSE was 0.209 m and the mean height RMSE was 0.058 m across the four trials. Considering the meter-level uncertainty of the GPS reference itself, these results show that the proposed method tracks the small residual motion of a hovering UAV consistently with the independent GPS reference, confirming its short-term stability.

Overall, the field measurements show that the proposed CECT-3DAL method provides good directional tracking of UAV acoustic sources in real, complex environments. Under hovering, the azimuth estimates remain stable within a 90% span of 2–3.5°, and registration to a GPS reference further yields an average horizontal RMSE of 0.209 m and an average height RMSE of 0.058 m across four trials; under straight, oblique, and circling flight, the estimated direction-vector trajectories are continuous and smooth and agree with the actual flight modes of the UAV. These results demonstrate that the proposed method can achieve stable direction estimation and trajectory tracking of low-altitude, slow-moving UAVs under outdoor conditions with strong noise and multiple disturbances, confirming its engineering practicality.

## 4. Discussion

The three-tier validation framework, consisting of simulation, anechoic chamber, and field experiments, provides a consistent and mutually corroborating picture of the proposed CECT-3DAL method. In the simulation, the azimuth and elevation errors decreased monotonically with increasing SNR and integration time, which directly confirms that the adaptive diagonal loading and the sliding-average covariance estimation work as intended. Both act to stabilize the covariance estimate, so that more signal energy or more snapshots translate into a more reliable subspace and thus a sharper, more accurate spectral peak. A consistent secondary observation across both the simulation and the field data is that the azimuth error was slightly larger than the elevation error. This is not incidental but a direct consequence of the AVS measurement geometry: at higher elevation angles, the horizontal velocity components scale with cos φ and therefore shrink, reducing the observability of the azimuth. The agreement between this predicted trend and the measured errors lends physical credibility to the estimator.

The anechoic chamber results further isolate the contribution of the temporal tracking stage. Confidence-weighted smoothing reduced the azimuth and elevation standard deviations from 4.34° and 2.63° to 1.46° and 0.86°, respectively—improvements that cannot be attributed to smoothing alone, since the gain stems specifically from down-weighting the low-confidence frames identified by the eigenvalue ratio. The EVR therefore acts as an effective reliability indicator, allowing transient, interference-corrupted frames to be suppressed without distorting the genuine direction trend. In the field, where wind, ambient noise, and ground reflections coexist, the trajectories reconstructed by the method remained consistent with the independent GPS reference, with hovering horizontal and height RMSEs of 0.209 m and 0.058 m. Notably, these residuals are of the same order as—or smaller than—the meter-level positioning uncertainty of the GPS reference itself, indicating that the observed discrepancy is dominated by the reference rather than by the acoustic estimator, and that the method retains its accuracy when transferred from controlled to realistic conditions.

When placed in the context of representative acoustic UAV localization studies, Blanchard et al. [20] achieved three-dimensional localization and tracking using an array with several spatially separated microphones, harmonic extraction, and Kalman filtering. Itare et al. [19] employed an optimized ten-microphone array with an aperture of up to 1.2 m for long-range UAV DOA tracking, whereas Lim et al. [25] used two distributed arrays comprising eight microphones to estimate the three-dimensional UAV position. In comparison, the proposed method requires only a single compact AVS with four collocated pressure–velocity channels and achieves azimuth and elevation RMSEs below 1.5° when the SNR exceeds 4 dB. In the outdoor hovering experiments, the acoustic trajectory registered to the GPS reference yielded an average horizontal RMSE of 0.209 m. Although these numerical results are not directly interchangeable because of differences in UAV type, measurement distance, acoustic environment, reference accuracy, and evaluation metrics, the comparison indicates that the proposed method provides competitive direction estimation and tracking performance with substantially reduced sensor count, aperture, and deployment complexity.

These results also clarify why an AVS-based scheme is attractive for this task. A single AVS acquires the acoustic pressure and three-axis particle velocity at one point, so directional information is obtained without the large aperture and many elements required by conventional microphone arrays, greatly reducing system size and deployment complexity. The adaptive diagonal loading and exponential spectral enhancement jointly improve the reliability of peak extraction under complex noise, while the EVR-based confidence weighting adds temporal robustness that a purely per-frame estimator lacks. Compared with radar, optical, and radio-frequency techniques, the acoustic approach is fully passive, unaffected by illumination and weather, and remains effective against UAVs that fly autonomously without a communication link—properties that make it a useful complement to, or fallback for, existing counter-UAV modalities.

## 5. Conclusions

To address the limited accuracy, large system size, and insufficient robustness of UAV acoustic localization in complex environments, this paper proposes an AVS-based method, termed Covariance Enhancement and Confidence-guided Tracking for 3D Acoustic Localization (CECT-3DAL). Using a single AVS to acquire the sound pressure and three-axis particle velocity simultaneously at a single point, the method employs adaptive diagonal loading to enhance the robustness of the covariance matrix at low SNR, applies exponential spectral enhancement to increase the contrast of the spectral peaks, and constructs an eigenvalue ratio-based localization confidence to perform confidence-weighted smoothing of the azimuth and elevation sequences. A dual-sensor geometric model is further derived to provide a closed-form solution for the 3D position of the source. Simulation, anechoic chamber, and field experiments show that the RMSEs of azimuth and elevation were below 1.5° when the SNR exceeded 4 dB. Confidence-weighted smoothing reduced the standard deviations of azimuth and elevation for the fixed source in the anechoic chamber from 4.34° and 2.63° to 1.46° and 0.86°, respectively. In the field, the azimuth estimates remained stable within a 90% span of 2–3.5° during hovering, and registration to a GPS reference further yielded an average horizontal RMSE of 0.209 m. Across four independent hover trials, the horizontal RMSE remained within 0.173–0.271 m and the height RMSE within 0.032–0.096 m, indicating good repeatability under hovering conditions. Continuous and smooth direction tracking was also achieved for straight, oblique, and circling flights. These results demonstrate that the proposed method achieves accurate and stable direction estimation and trajectory tracking of low-altitude, slow-moving UAVs in a compact, passive, and low-cost manner.

This study has several limitations. A single AVS provides only directional information. Although a closed-form dual-sensor 3D model was derived, independent 3D positioning was not experimentally validated, and the field study relied on a GPS reference of only meter-level accuracy. In addition, the method assumes far-field, relatively high-SNR conditions, and has not yet been compared with baseline methods through ablation studies. Future work will deploy two or more synchronized AVSs for full 3D position tracking, add quantitative comparisons and ablation experiments against classical methods, and introduce motion models and multi-source separation to handle maneuvering targets and multi-UAV scenarios so as to extend the approach toward a practical counter-UAV acoustic surveillance system for sensitive areas such as airports.

## Figures and Tables

**Table 1:** Source-to-sensor distances and estimated SNRs for the two anechoic chamber measurements.

Case	Source-to-Sensor Distance	Estimated SNR
Near-distance case	r1 = 0.85 m	16.4 dB
Far-distance case	r2 = 1.70 m	19.3 dB

## Data Availability

Data available on request due to privacy or ethical restrictions. The data presented in this study are available on request from the corresponding author.
